# Brevibacillin 2V Exerts Its Bactericidal Activity *via* Binding to Lipid II and Permeabilizing Cellular Membranes

**DOI:** 10.3389/fmicb.2021.694847

**Published:** 2021-07-16

**Authors:** Xinghong Zhao, Xiaoqi Wang, Rhythm Shukla, Raj Kumar, Markus Weingarth, Eefjan Breukink, Oscar P. Kuipers

**Affiliations:** ^1^Department of Molecular Genetics, Groningen Biomolecular Sciences and Biotechnology Institute, University of Groningen, Groningen, Netherlands; ^2^Membrane Biochemistry and Biophysics, Bijvoet Centre for Biomolecular Research, Department of Chemistry, Faculty of Science, Utrecht University, Utrecht, Netherlands; ^3^NMR Spectroscopy, Bijvoet Centre for Biomolecular Research, Department of Chemistry, Faculty of Science, Utrecht University, Utrecht, Netherlands

**Keywords:** mode of action, lipopeptide, non-ribosomally produced peptides, brevibacillin 2V, Lipid II, NMR

## Abstract

Lipo-tridecapeptides, a class of bacterial non-ribosomally produced peptides, show strong antimicrobial activity against Gram-positive pathogens, including antibiotic-resistant *Staphylococcus aureus* and *Enterococcus* spp. However, many of these lipo-tridecapeptides have shown high hemolytic activity and cytotoxicity, which has limited their potential to be developed into antibiotics. Recently, we reported a novel antimicrobial lipo-tridecapeptide, brevibacillin 2V, which showed no hemolytic activity against human red blood cells at a high concentration of 128 mg/L, opposite to other brevibacillins and lipo-tridecapeptides. In addition, brevibacillin 2V showed much lower cytotoxicity than the other members of the brevibacillin family. In this study, we set out to elucidate the antimicrobial mode of action of brevibacillin 2V. The results show that brevibacillin 2V acts as bactericidal antimicrobial agent against *S. aureus* (MRSA). Further studies show that brevibacillin 2V exerts its bactericidal activity by binding to the bacterial cell wall synthesis precursor Lipid II and permeabilizing the bacterial membrane. Combined solid-state NMR, circular dichroism, and isothermal titration calorimetry assays indicate that brevibacillin 2V binds to the GlcNAc-MurNAc moiety and/or the pentapeptide of Lipid II. This study provides an insight into the antimicrobial mode of action of brevibacillin 2V. As brevibacillin 2V is a novel and promising antibiotic candidate with low hemolytic activity and cytotoxicity, the here-elucidated mode of action will help further studies to develop it as an alternative antimicrobial agent.

## Introduction

**Figure 1:**
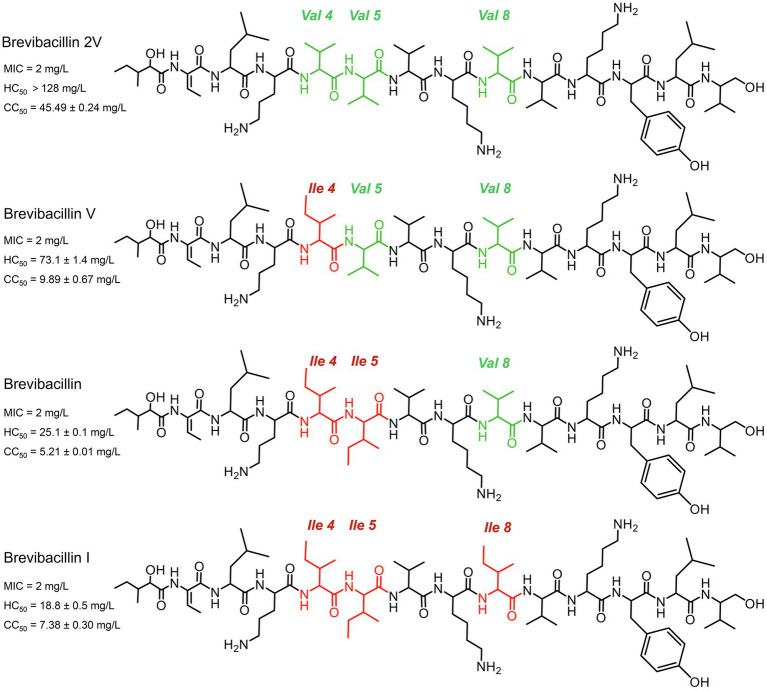
Structures of brevibacillins. The red color indicates the more hydrophobic amino acid residues of brevibacillin V, brevibacillin and brevibacillin I relative to brevibacillin 2V. MIC, the minimum inhibitory concentration against *Staphylococcus aureus* (MRSA). HC_50_, the 50% human blood cell hemolysis concentration. CC_50_, the 50% cell (HepG2) toxicity concentration.

A vast number of bacterial non-ribosomally produced peptides (NRPs) have shown strong antimicrobial activity against pathogenic bacteria (Cochrane et al., 2014; Cociancich et al., 2015; Hamamoto et al., 2015; Ling et al., 2015; Süssmuth and Mainz, 2017). Lipo-tridecapeptides, a class of NRPs, show strong antimicrobial activity against Gram-positive pathogens, including antibiotic-resistant *Staphylococcus aureus* and *Enterococcus* spp. (Barsby et al., 2001, 2006; Yang et al., 2016; Wu et al., 2019; Zhao et al., 2021). However, many of these lipo-tridecapeptides have shown high hemolytic activity and cytotoxicity, which limits their potential to be developed into antibiotics (Li et al., 2018; Zhao et al., 2021). Recently, several lipo-tridecapeptides (brevibacillins) were discovered from *Brevibacillus laterosporus* DSM 25 by genome mining (Figure 1), which all showed similar antimicrobial activity against the tested Gram-positive pathogenic bacteria (Zhao et al., 2021). In contrast to other brevibacillins, one of the novel lipo-tridecapeptides, brevibacillin 2V, showed no hemolytic activity against human red blood cells at a high concentration of 128 mg/L (Zhao et al., 2021). In addition, brevibacillin 2V showed much lower cytotoxicity than the other brevibacillins (Figure 1). These properties make brevibacillin 2V a promising candidate for developing as an alternative antibiotic to control specific antibiotic-resistant bacterial pathogens. Although brevibacillin has been reported 5 years ago (Yang et al., 2016), little is known about its antimicrobial mode of action. The only available information is that brevibacillin can permeabilize the membrane of Gram-positive bacteria (Yang et al., 2017). As brevibacillin 2V is a promising antibiotic candidate, elucidation of its antimicrobial mode of action would help the potential application development.

**Figure 2:**
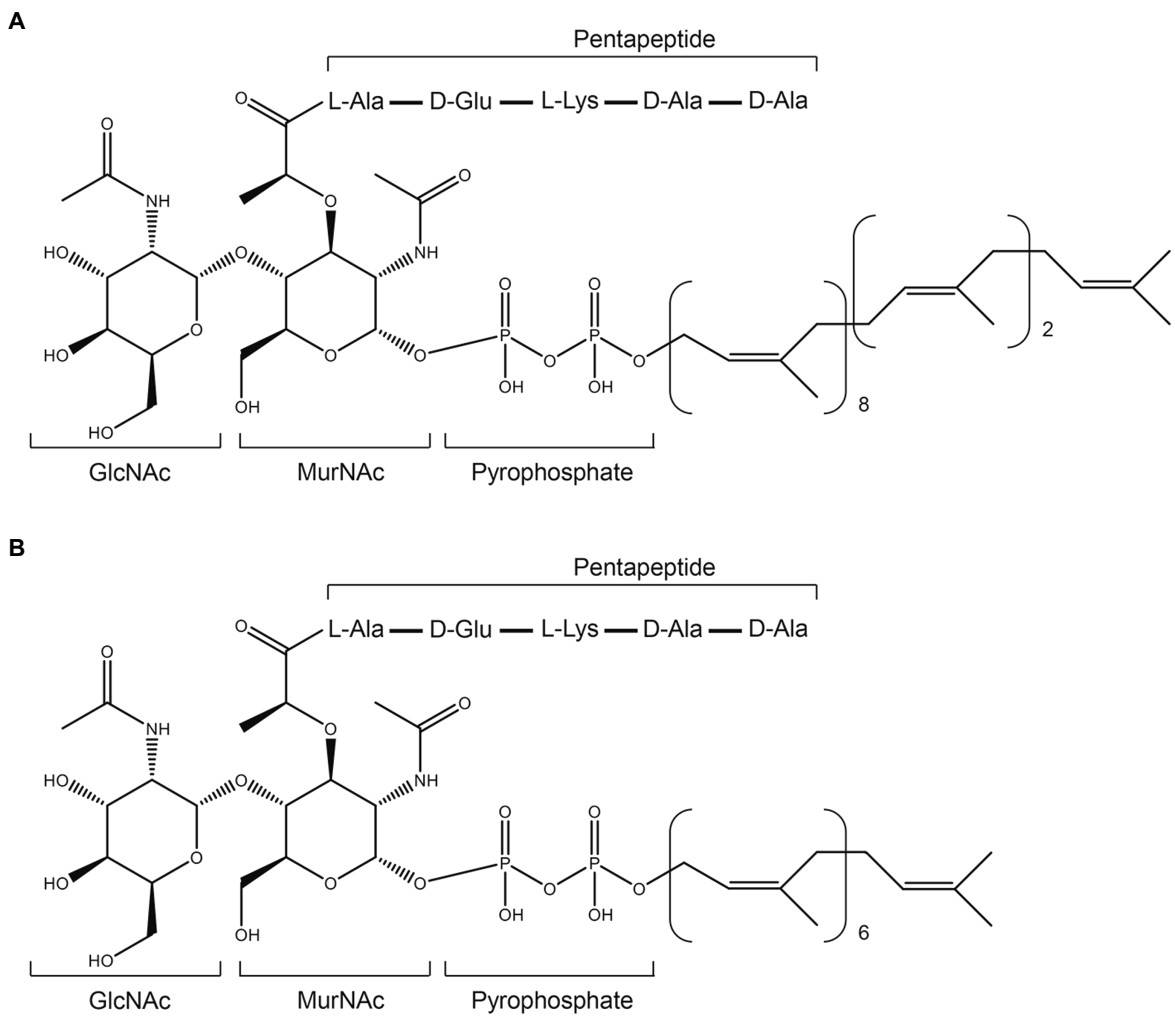
Structures of Gram-positive Lipid II and its variant. **(A)**, structure of Gram-positive Lipid II. **(B)**, the heptaprenyl version of Gram-positive Lipid II.

Lipid II (GlcNAc-MurNAc-pentapeptide-pyrophosphoryl-undecaprenol; Figure 2A) is an essential precursor for synthesizing the bacterial cell wall (Hsu et al., 2004; Breukink and de Kruijff, 2006). The vital role of Lipid II in cell wall synthesis makes it an excellent target for many antibiotics, including vancomycin, ramoplanin, mannopeptimycins, nisin, NAI-107, gallidermin, and include two recently found lipopeptides: teixobactin and tridecaptin A1 (Breukink et al., 1999; Hsu et al., 2004; Breukink and de Kruijff, 2006; Münch et al., 2014; Ling et al., 2015; Cochrane et al., 2016). These antibiotics bind to Lipid II and thereafter cause abduction of Lipid II from the cell wall synthesis sites (Hasper et al., 2006).

In this study, we aimed to elucidate the antimicrobial mode of action of brevibacillin 2V. First, a time-killing assay was performed to investigate whether brevibacillin 2V acts either as a bacteriostatic or bactericidal agent. The results demonstrate that brevibacillin 2V is a bactericidal antimicrobial agent against *S. aureus* (MRSA). Next, 3,3´-dipropylthiadicarbocyanine iodide [DiSC_3_(5)], carboxyfluorescein (CF) leakage, and fluorescence microscopy assays show that brevibacillin 2V has a membrane permeabilization capacity. Additionally, spot-on-lawn, circular dichroism, fluorescence quenching, and isothermal titration calorimetry (ITC) assays demonstrated that brevibacillins display antimicrobial activity by targeting the essential cell wall synthesis precursor Lipid II. Finally, combined solid-state NMR (ssNMR), circular dichroism, and ITC assays indicate that brevibacillin 2V binds to the GlcNAc-MurNAc moiety and/or the pentapeptide of Lipid II, in contrast to teixobactin, nisin, and other peptides that target the pyrophosphate moiety.

## Materials and Methods

### Bacterial Strains Used and Growth Conditions

*Brevibacillus laterosporus* DSM 25 cells were inoculated in LB and incubated at 37°C with shaking at 220 rpm for preparing overnight culture. For the production of brevibacillins, an overnight culture of *B. laterosporus* DSM 25 cells was inoculated (50-fold dilution) in minimal expression medium and grown for 36 h at 30°C with shaking at 220 rpm. Subsequently, brevibacillins were purified as described in the previous studies (Zhao et al., 2021; Zhao and Kuipers, 2021). *S. aureus* ATCC15975 (MRSA) was inoculated on LB and incubated at 37°C with shaking at 220 rpm for preparing the overnight cultures.

### Lipid II and Its Variant Preparation

Lipid II and 7-Lipid II (a heptaprenyl version of Lipid II) were synthesized and purified as described in a previous study (Breukink et al., 2003; Figures 2A,B). Purified Lipid II and 7-Lipid II were dissolved in CHCl_3_/MeOH (2:1) and stored at −20°C until use, and the concentration was determined after destruction to inorganic phosphate according to Rouser et al. (1970). 7-Lipid II was only used in circular dichroism assays, while wild-type Lipid II was used in all of the other assays.

### Time-Killing Assay

This assay was performed according to a previously described procedure (Ling et al., 2015; Zhao et al., 2020b). An overnight culture of cells [*S. aureus* ATCC15975 (MRSA)] was diluted 50-fold in MHB and incubated at 37°C with aeration at 220 rpm. Bacteria were grown to an OD_600_ of 0.5, and then, the concentration of cells was adjusted to ≈ 5 × 10^6^ cells per ml. Bacteria were then challenged with nisin (40 μg/ml), brevibacillin (20 μg/ml), brevibacillin V (20 μg/ml), brevibacillin I (20 μg/ml), or brevibacillin 2V (20 μg/ml) in culture tubes at 37°C and 220 rpm (peptides at 10 × MIC, a desirable concentration at the site of infection). Bacteria not treated with peptides were used as negative controls. At desired time points, 200 μl aliquots were taken, centrifuged at 8,000 g for 2 min, and resuspended in 200 μl of MHB. 10-fold serially diluted samples were plated on MHA plates. After incubated at 37°C overnight, colonies were counted and c.f.u. per ml was calculated. Each experiment was performed in triplicate.

### 3,3´-Dipropylthiadicarbocyanine Iodide Assay

*Staphylococcus aureus* ATCC15975 (MRSA) was grown to an OD_600_ of 0.8. The culture was pelleted at 5,000 × *g* for 5 min and washed three times in 10 mm HEPES with 10 mm glucose (pH 7.2). The cell density was normalized to an OD_600_ of 0.2, loaded with 2 μm DiSC_3_(5) dye, and incubated for 20 min in the dark for probe fluorescence to stabilize. After incubation, KCl was added to the cell suspension at a final concentration of 100 mm. Subsequently, the cell suspension was added to a 96-well microplate and incubation for 5 min; after that, antibiotics were added at a final concentration of 2 × MIC. Fluorescence was monitored for 25 min, with the antibiotics added after ~20 s. The excitation and emission wavelengths on the fluorescence spectrometer were adjusted to 622 nm and 670 nm, respectively. Representative examples from three technical replicates are shown.

### Microscopy Assay

*Staphylococcus aureus* ATCC15975 (MRSA) was grown to an OD_600_ of 0.8. The culture was pelleted at 5,000 × *g* for 5 min and washed three times in 10 mm HEPES with 10 mm glucose (pH 7.2). After normalization of the cell density to an OD_600_ of 0.2 in 10 mm HEPES with 10 mm glucose (pH 7.2), a 2-fold MIC concentration of brevibacillins (4 μg/ml) or nisin (8 μg/ml) was added. At the same time, SYTO^®^ 9 and propidium iodide (LIVE/DEAD Baclight Bacterial Viability Kit, Invitrogen) were added to the above cell suspensions. After incubation at room temperature for 15 min, peptides were removed and washed three times with 10 mm HEPES with 10 mm glucose (pH 7.2). Then, the cell suspensions were loaded on 1.5% agarose pads and analyzed by DeltaVision Elite microscope (Applied Precision; Zhao and Kuipers, 2021).

### Spot-on-Lawn Assay

An overnight cultured *S. aureus* ATCC15975 (MRSA) was added to 0.6% MHA (w/v, temperature 42°C) at a final concentration of 0.1% (v/v), and then, the mixture was poured to the plates 10 ml for each. The binding of peptide and Lipid II (Hasper et al., 2006) was further evaluated by spotting of purified Lipid II (300 μm, 4 μl) to the edge of inhibition halo of antibiotics (brevibacillins, 0.68 μg; nisin and daptomycin, 1.36 μg). Briefly, antibiotics were loaded to the agar plate. After the antibiotic solution drops had dried, purified Lipid II was spotted to the edge (identified by a pre-experiment) of inhibition halo of antibiotics (Wang et al., 2020). After the Lipid II solution drops had dried, the plates were transferred to a 37°C incubator for overnight incubation.

### Circular Dichroism Assay

7-Lipid II and 1,2-dioleoyl-sn-glycero-3-phosphocholine (DOPC) were resuspended in 10 mm boric acid-NaOH pH 7.5 containing 0.1% CHAPS. The final concentration of antibiotics and 7-Lipid II was adjusted to 50 μm, while a 100 μm final concentration of DOPC vesicles was used. After added the samples to a quartz cuvette with a path length of 1 mm, the CD spectra were recorded with a Jasco-810 CD spectropolarimeter at 20°C. The ellipticity was recorded between 185 nm and 260 nm at a 0.2-nm step size with 1-s response time. The spectra were averaged over five recordings with a scanning speed of 50 nm/min.

### Fluorescence Quenching Assay

The effects of antibiotics on pyrene-labeled Lipid II were measured using 50 μm DOPC-based large unilamellar vesicles (LUVs) containing 0.5 mol % pyrene-labeled Lipid II (Breukink et al., 2003). Briefly, the LUVs were added to a quartz cuvette and titrated with nisin, brevibacillin, or brevibacillin 2V with stirring at 20°C. The fluorescence spectra were recorded between 360 and 600 nm (λ_ex_350 nm) using a Cary Eclipse Fluorescence Spectrophotometer (Agilent, United States).

### Isothermal Titration Calorimetry Assay

LUVs containing Lipid II (2%, mol/mol) were prepared by mixing the appropriate volumes of Lipid II and DOPC stock solutions in CHCl_3_/MeOH (2:1, v/v). The lipid solutions were dried by a nitrogen stream and hydrated with 10 mm Tris-HCl, 100 mm NaCl, and pH 8 buffer to a lipid-phosphate concentration of ~ 20 mm. LUVs were obtained after 10 times freeze-thaw cycles followed by 10 rounds of extrusion through 200 nm membrane filters (Whatman Nuclepore, Track-Etch Membranes). The concentration of lipid-phosphate was determined as described (Rouser et al., 1970).

Isothermal titration calorimetry was performed with the Low Volume Nano ITC (Waters LLC, New Castle, DE, United States) to determine the interaction between LUVs and brevibacillins. Brevibacillins were diluted in a buffer (10 mm Tris-HCl, 100 mm NaCl, and pH 8) to a final concentration of 50 μm. Samples were degassed before use. The chamber was filled with 177 μl of the brevibacillins solutions, and the LUVs were titrated into the chamber at a rate of 2 μl/300 s with a stirring rate of 300 rpm. Experiments were performed at 25°C. Control experiments were performed with Lipid II-free LUVs. The Kd values of brevibacillins to Lipid II were calculated using the Nano Analyze Software (Waters LLC).

### Carboxyfluorescein Leakage Assay

LUVs that contain DOPC or DOPC plus 0.1% Lipid II were prepared for a carboxyfluorescein (CF) leakage assay. The lipids were dried by a nitrogen stream and followed under vacuum for 2 h. After that, the lipids were hydrated by adding 25 mm Tris-HCl, 150 mm NaCl, and pH 7.5 containing 50 mm CF. The suspensions were frozen and thawed 10 times, followed by extrusion through 200nm membrane filters (Whatman Nuclepore, Track-Etch Membranes) 10 times. The excess dye was removed by loading vesicles on a spin column (Sephadex G50) for 2 min at 500 × *g*. Subsequently, the vesicles were diluted to 5 μm, and the brevibacillins-induced release of CF from the vesicles was monitored by measuring the increase in fluorescence intensity. The maximum fluorescence was reached by adding 10 μl of 20% Triton X-100. A Cary Eclipse Fluorescence Spectrophotometer (Agilent, United States) was used to determine the changes of fluorescence signals, and the excitation wavelength and emission wavelength were adjusted to 492 nm and 515 nm, respectively.

### Solid-State NMR Spectroscopy Assay

Multi-lamellar vesicles of DOPC doped with 2 mol % Lys-Lipid II in buffer (20 mm HEPES, 100 mm NaCl, and pH = 8) were collected by centrifugation (60,000 × *g*) and loaded into 3.2 mm ssNMR rotors. For 3.2 mm rotors, we used 200 nmol of antibiotic with 100 nmol of Lipid II. The 1D ^31^P ssNMR experiments were performed at 500 MHz (^1^H frequency) magnetic field using 10 kHz magic angle spinning (MAS) at 270 K sample temperature.

### Quantification and Statistical Analyses

**Figure 3:**
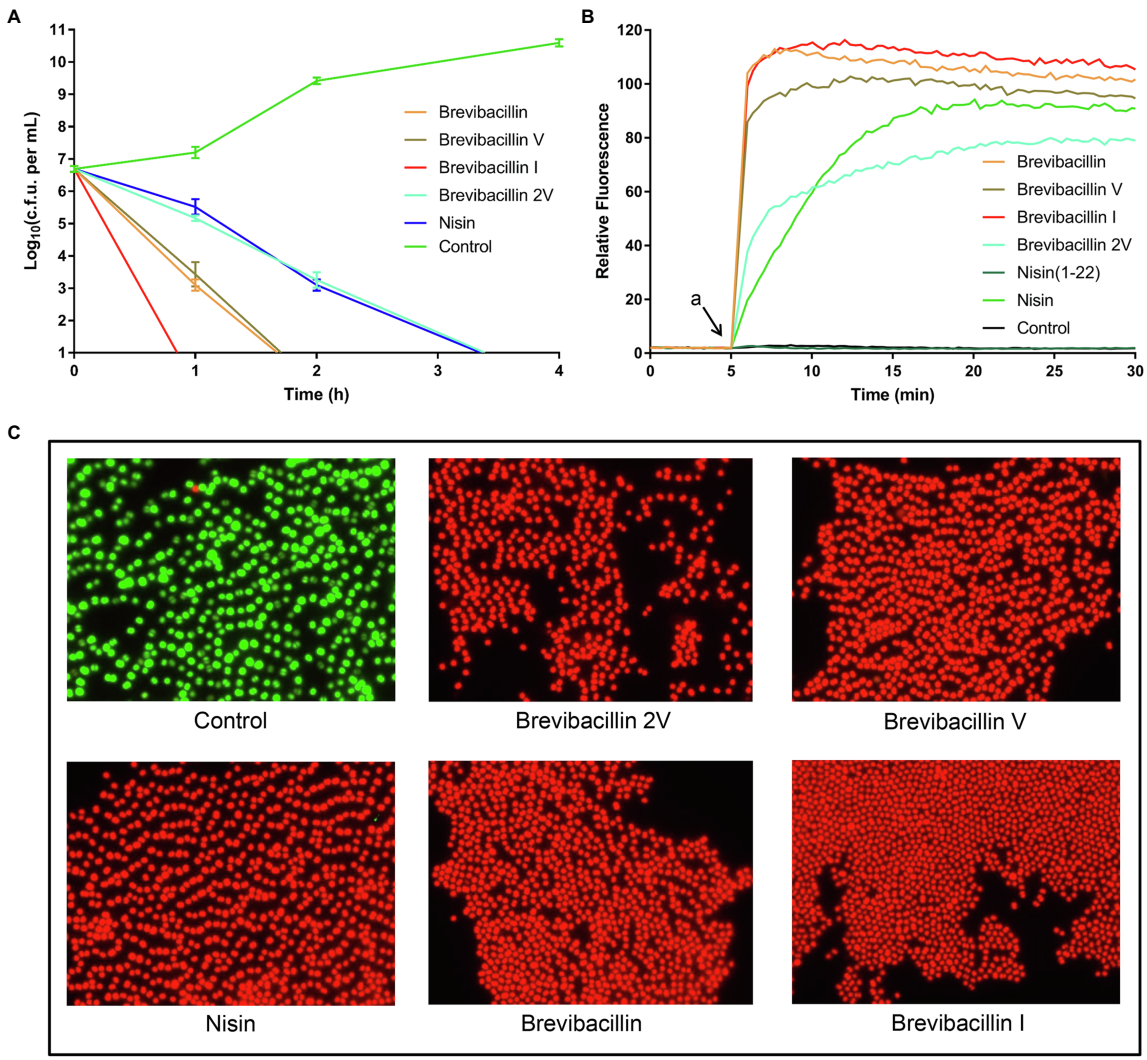
Brevibacillins disrupt the cellular membrane and act as bactericidal antibiotics. **(A)**, time-killing curves of brevibacillins (10 × MIC) against *S. aureus* (MRSA). **(B)**, DiSC_3_(5) fluorescence in *S. aureus* (MRSA) upon exposure (at 2 × MIC) to brevibacillins, nisin, nisin (1-22), and MQ (Control); a, when peptides were added. **(C)**, fluorescence microscopic images of *S. aureus* (MRSA) treated by brevibacillins and nisin at a concentration of 2 × MIC for 15 min.

**Figure 4:**
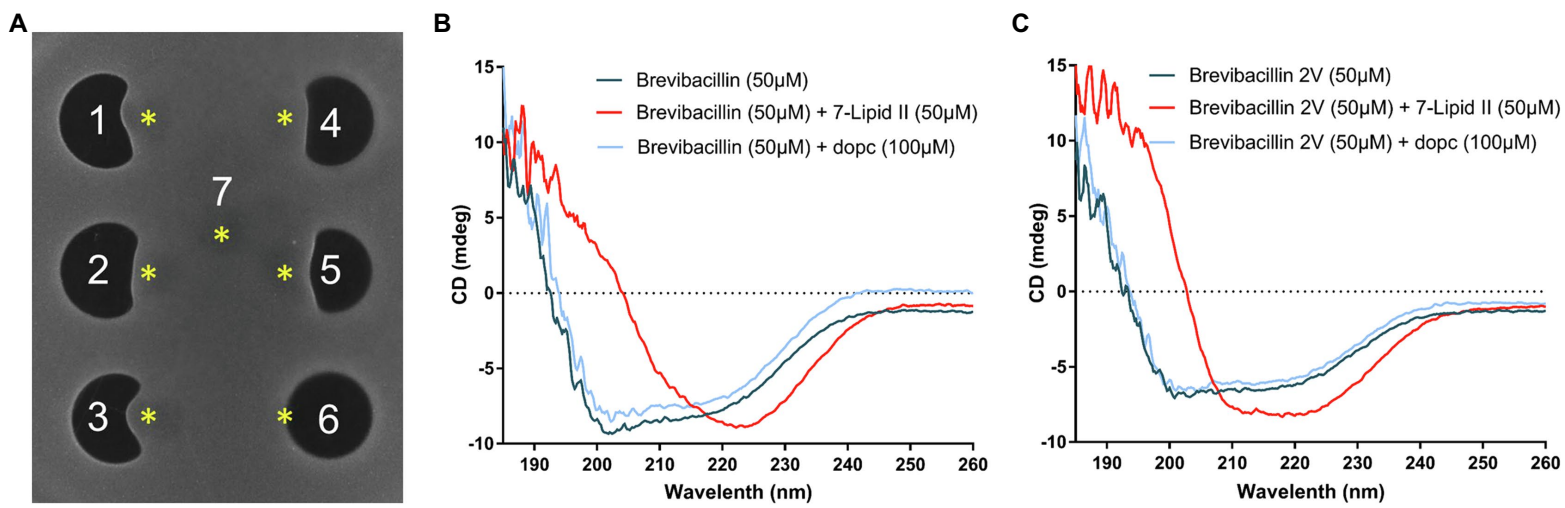
Brevibacillins bind to the cell wall synthesis precursor Lipid II. **(A)**, Spot-on-lawn assay with *S. aureus* (MRSA). 1, brevibacillin; 2, brevibacillin V; 3, brevibacillin I; 4, brevibacillin 2V; 5, nisin; 6, daptomycin; and 7, MQ. Brevibacillins were added at a concentration of 100 μg/ml and with a volume of 6.8 μl; nisin and daptomycin were added at a concentration of 200 μg/ml and with a volume of 6.8 μl. ^*^, The position of Lipid II added (300 μm, 4 μl). **(B)**, CD spectrum of brevibacillin, brevibacillin plus 7-Lipid II, and brevibacillin plus DOPC. **(C)**, CD spectrum of brevibacillin 2V, brevibacillin 2V plus 7-Lipid II, and brevibacillin 2V plus DOPC.

**Figure 5:**
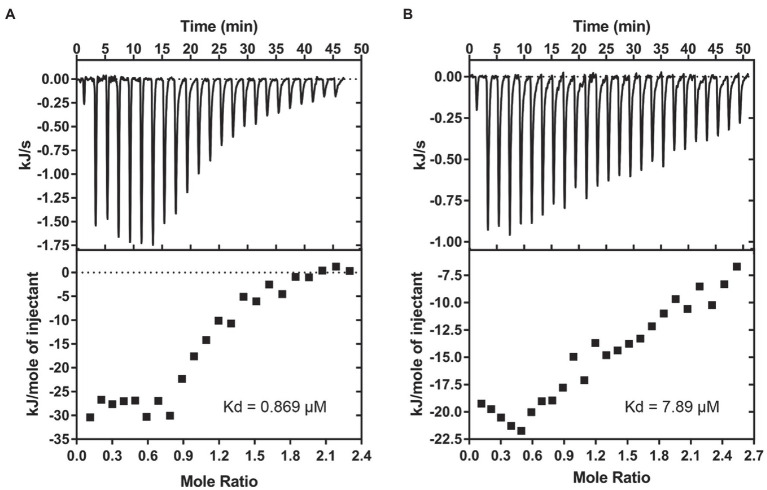
Brevibacillins show good affinity to Lipid II. **(A)**, ITC-binding experiments of brevibacillin and phospholipid LUVs containing 2 mol % Lipid II. **(B)**, ITC-binding experiments of brevibacillin 2V and phospholipid LUVs containing 2 mol % Lipid II. The Kd values of brevibacillins to Lipid II were calculated using the Nano Analyze Software (Waters LLC).

**Figure 6:**
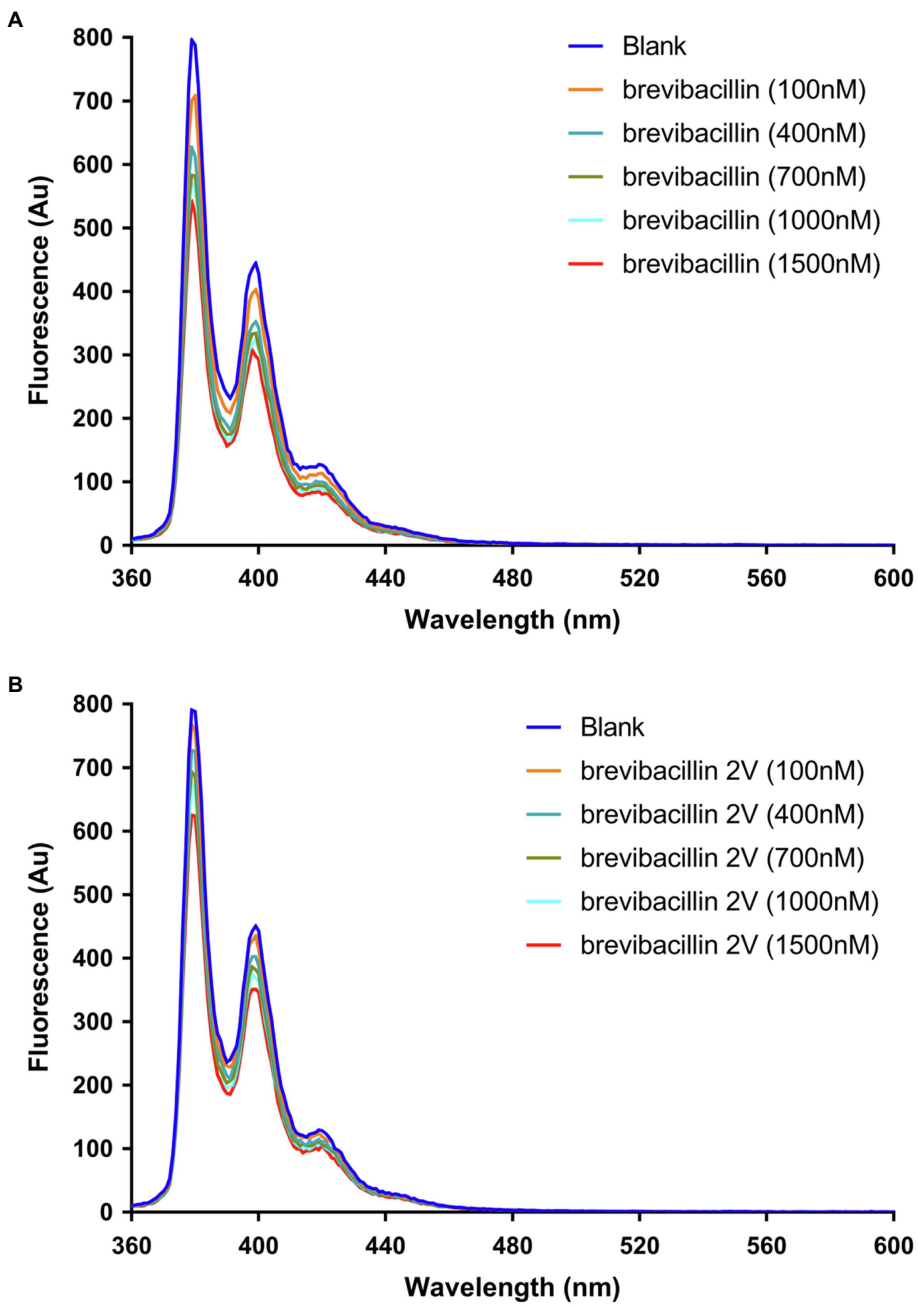
Brevibacillins quench the fluorescence of pyrene-labeled Lipid II. **(A)**, Effect of increasing brevibacillin concentrations on the fluorescence characteristics of pyrene-labeled Lipid II at 0.5 mol % in DOPC bilayers. **(B)**, Effect of increasing brevibacillin 2V concentrations on the fluorescence characteristics of pyrene-labeled Lipid II at 0.5 mol % in DOPC bilayers.

**Figure 7:**
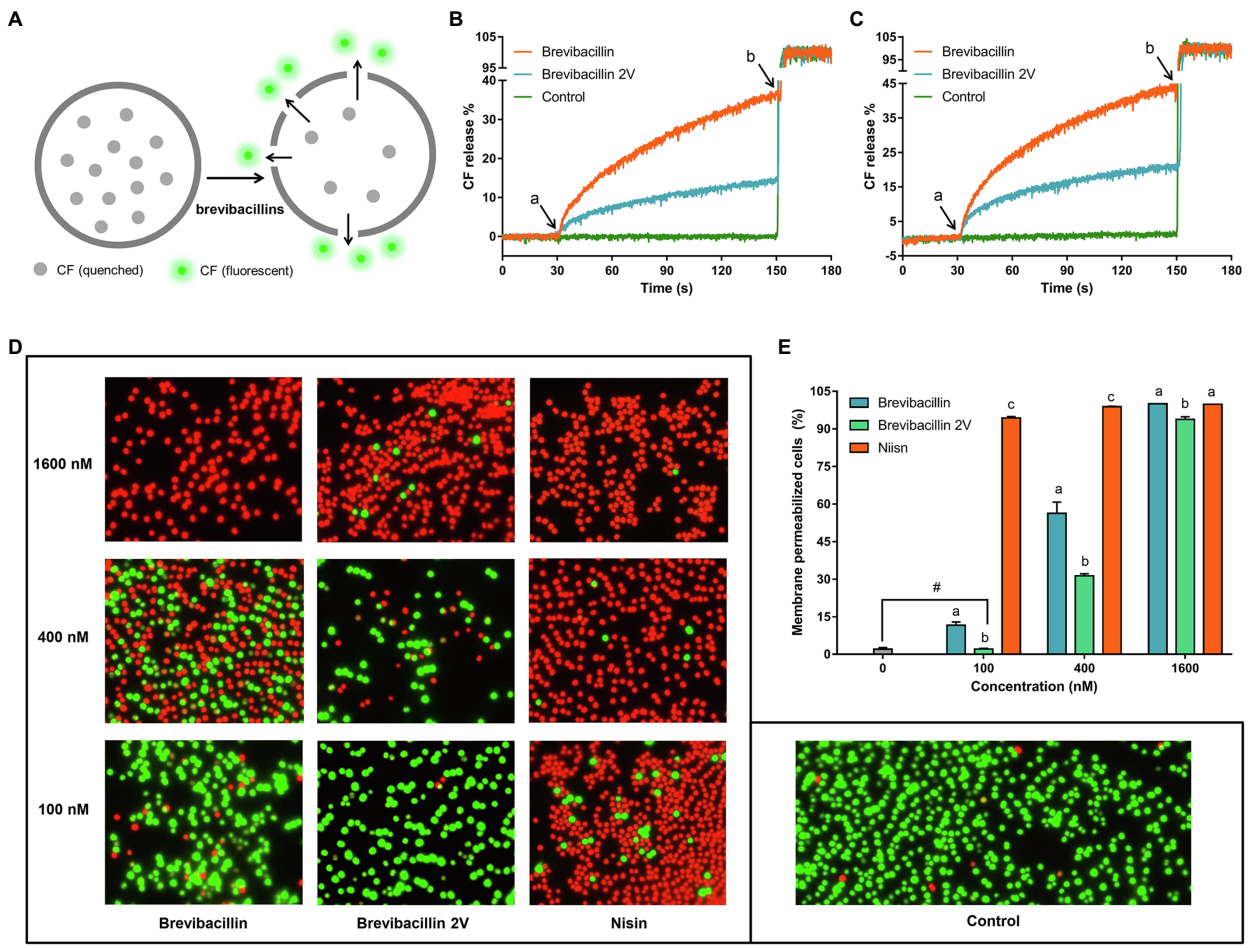
Brevibacillins disrupt membrane permeability without the need for Lipid II binding. **(A)**, Cartoon schematic of the assay used to assess membrane permeability disruption of brevibacillins. **(B)**, CF releases (%) vs. time graph showing the effects of adding brevibacillins to DOPC-LUVs with 0.1% Lipid II; peptide concentrations were 100 nm; a, when peptides were added; and b, when 20% Triton X-100 added. **(C)**, CF releases (%) vs. time graph showing the effects of adding brevibacillins to LUVs of pure DOPC; peptide concentrations were 100 nm; a, when peptides were added; and b, when 20% Triton X-100 was added. **(D)**, fluorescence microscopic images of *S. aureus* (MRSA) after have been treated by a series concentrations of brevibacillins and nisin for 15 min. **(E)**, the ratio of membrane permeabilized cells that was quantified by Adobe Photoshop 2021; the data are representative of three independent experiments. Significant differences were considered at *p* < 0.05; ^a,b,c^ bars at the same concentration without the same superscripts differ significantly (*p* < 0.05); and ^#^ brevibacillin 2V did not induce membrane permeabilization at a concentration of 100 nm, which showed no significant difference on the ratio of membrane permeabilized cells with untreated group.

GraphPad Prism 7.0 was used to fit the data of time-killing assay, DiSC_3_(5) assay, fluorescence quenching assay, ITC assay, and CF Leakage assay in Figures 3–7. The fluorescence images (ratio of membrane permeabilized cells) were quantified by the software Adobe Photoshop 2021. The Kd values of brevibacillins to Lipid II were calculated using the Nano Analyze Software (Waters LLC). The statistical significance of the data was assessed using Duncan’s multiple range test with the software SPSS Statistics 26; values of *p* < 0.05 were considered to be statistically significant.

## Results and Discussion

### Brevibacillins Disrupt the Cellular Membrane and Act as Bactericidal Antibiotics

The time-killing assay is a widely used method to assess whether an antibiotic is bacteriostatic or bactericidal (Ling et al., 2015; Cochrane et al., 2016; Zhao et al., 2020b). In the present study, we monitored the killing kinetics at 10 times the MIC value of brevibacillins against *S. aureus* (MRSA) cells, and nisin was used as a control for bactericidal antibiotic activity (Hasper et al., 2006). Nisin completely killed all cells within 4 h post-exposure, while brevibacillin I completely killed all cells within 1 h post-exposure, making it the fastest killing antibiotic in this study (Figure 3A). Brevibacillin and brevibacillin V completely killed all cells within 2 h post-exposure, showing an intermediate killing capacity (Figure 3A). Brevibacillin 2V displayed the slowest killing capacity among brevibacillins, with similar killing kinetics as nisin (Figure 3A). These results demonstrate that brevibacillins are fast-acting bactericidal antibiotics. Such a fast bactericidal activity suggests that these peptides target the bacterial membranes.

Indeed, many lipopeptide antibiotics have membrane disruption capacities, such as polymyxin B, daptomycin, brevicidine, iturins, and many others (Maget-Dana and Peypoux, 1994; Seydlová et al., 2019; Zhao et al., 2020a). To test membrane permeabilization, we used the membrane-potential sensitive dye DiSC_3_(5), which is widely used in the mode of action studies of antibiotics (Rink et al., 2007; Jangra et al., 2019; Valderrama et al., 2019; Stokes et al., 2020). DiSC_3_(5) accumulates in bacterial membranes that have a membrane potential and self-quenches its fluorescence (Wu et al., 1999; Stokes et al., 2020). When the membrane potential is dissipated due to membrane permeabilization, this probe is released from the membrane resulting in an increased fluorescence signal (Wu et al., 1999; Stokes et al., 2020). In the present study, we monitored the changes in membrane integrity of *S. aureus* in the presence of brevibacillins. Nisin and nisin (1-22) were used as antibiotic controls, i.e., with and without membrane disruption power, respectively. The membrane of *S. aureus* was quickly permeabilized by the exposure to all four individual brevibacillins (Figure 3B). In addition, the membrane disruption capacity of brevibacillins shows a positive correlation with their calculated hydrophobicity, i.e., brevibacillin I > brevibacillin > brevibacillin V > brevibacillin 2V (Figures 1, 3B; Zhao et al., 2021). These results suggest that the N-terminal region of brevibacillins plays a vital role in interacting with cellular membranes; a higher hydrophobic N-terminus of brevibacillins probably infers a higher membrane disruption capacity. In this assay, brevibacillin I, brevibacillin, and brevibacillin V appeared to act faster than brevibacillin 2V and the well-known pore-forming antibiotic nisin (Figure 3B), which is in line with the killing kinetics (Figure 3A).

To exclude that the membrane disruption ability of brevibacillins was caused by cell lysis, a fluorescence microscopy assay was performed by a commercial LIVE/DEAD Baclight Bacterial Viability Kit, which consists of SYTO^®^ 9 and propidium iodide. SYTO^®^ 9 is a membrane-permeable green-fluorescent nucleic acid stain, which stains virtually all cells (Stocks, 2004). Propidium iodide is a membrane-impermeable red-fluorescent nucleic acid stain, which cannot enter intact, healthy cells (Stocks, 2004). Cells with an intact membrane will stain green, whereas cells with a compromised membrane will stain red. *S. aureus* cells were treated with peptides (2 × MIC) for 15 min in the presence of SYTO^®^ 9 and propidium iodide and then analyzed by a DeltaVision Elite microscope (Applied Precision). Green cells were observed for the non-antibiotic treated cells since they had intact membranes (Figure 3C). All brevibacillin- and nisin-treated cells turned red, indicative of a compromised membrane (Figure 3C). These results are consistent with the results of the DiSC_3_(5) assays. In addition, no cell lysis was observed from the microscopic images, which demonstrates that the membrane disruption ability of brevibacillins is not leading to rapid lysis. These results suggest that the capacity of brevibacillins to permeabilize the membrane of target cells contributes to their bactericidal activity.

### Brevibacillins Bind to the Cell Wall Synthesis Precursor Lipid II

Lipid II is an essential precursor for bacteria cell wall synthesis (Hsu et al., 2004; Breukink and de Kruijff, 2006). Nisin is a well-known Lipid II-targeting antibiotic (Breukink et al., 1999; Breukink and de Kruijff, 2006), and it was used as a Lipid II-targeting antibiotic control. As brevibacillins show a similar membrane permeability capacity as nisin and display a similar antimicrobial activity as nisin against both Gram-positive and Gram-negative bacterial pathogens, we investigated the Lipid II-binding capacity of brevibacillins by an agar diffusion assay similar to the method described in a previous study (Wang et al., 2020; Figure 4A). Due to binding to externally added purified Lipid II, the antimicrobial activity of nisin against *S. aureus* (MRSA) was diminished, resulting in a disruption of the normally round antibiotic-induced halo. Daptomycin was used as a non-Lipid II-binding antibiotic (as this was done in the absence of phosphatidylglycerol), which kept its antimicrobial activity against *S. aureus* (MRSA) upon the addition of purified Lipid II, resulting in a circular halo. Surprisingly, brevibacillins also lost their antimicrobial activity against *S. aureus* (MRSA) by the addition of purified Lipid II (Figure 4A). These results suggest that Lipid II is a target for brevibacillins to exert their antimicrobial activity. As brevibacillin 2V showed no hemolytic activity and brevibacillin had the highest production level (Zhao et al., 2021), these two compounds were selected for further studies. To further investigate the brevibacillins and their putative Lipid II binding, circular dichroism (CD) spectra of brevibacillin and brevibacillin 2V together with the heptaprenyl version of Lipid II (7-Lipid II) or DOPC were monitored. For both peptides, a significant conformational change could be observed in the presence of 7-Lipid II, while no change was apparent in the presence of DOPC (Figures 4B,C; Supplementary Figure 1). These results demonstrate that both brevibacillin and brevibacillin 2V can directly bind to the cell wall synthesis precursor Lipid II.

### Brevibacillin and Brevibacillin 2V Show Good Affinity to Lipid II

After confirmation of the fact that brevibacillins bind to Lipid II, an ITC assay was performed to determine the binding affinity of brevibacillins for Lipid II. For this, 2 mol % Lipid II was incorporated into DOPC-based LUVs. Brevibacillin showed a precise binding curve with a solid affinity to membrane-embedded Lipid II, from which a Kd value of 0.9 μm could be calculated (Figure 5A). In addition, brevibacillin 2V also showed binding to membrane-embedded Lipid II, albeit with somewhat lower affinity with a Kd value of 7.9 μm (Figure 5B). Nisin, teixobactin, nukacin ISK-1, and lacticin 3147 are well-known Lipid II-binding antibiotics; among these antibiotics, nisin showed the strongest binding affinity to membrane-embedded Lipid II with a Kd value of 14.6 nm, and lacticin 3147 showed the lowest binding affinity to membrane-embedded Lipid II with a Kd value of 0.92 μm (Islam et al., 2012; ‘t Hart et al., 2016; Bakhtiary et al., 2017; Chiorean et al., 2020). In this study, brevibacillins showed solid-binding affinities (Kd, 0.9 μm to 7.9 μm), and they were reported to have comparable antimicrobial activities as nisin and lacticin 3147 against the tested bacterial pathogens (Zhao et al., 2021). These results may indicate that an additional factor is involved in obtaining optimal-binding affinity of Lipid II and these types of antimicrobial peptides *in vivo*.

### Brevibacillins Quench the Fluorescence of Pyrene-Labeled Lipid II

It has been shown using pyrene-labeled Lipid II that nisin assembles together with Lipid II into a pore-complex (Breukink et al., 2003). We tested whether this is also the case for the brevibacillins. Pyrene monomers have characteristic fluorescence emission maxima at about 378 nm, 398 nm, and 417 nm. In addition, pyrene-Lipid II can display a unique fluorescence emission maximum at about 490 nm, when two different pyrenes residues form an excited state dimer (excimer; Breukink et al., 2003). As a control, we first used nisin, which clearly induced a decrease of pyrene monomer fluorescence that coincided with the appearance of a fluorescence peak around 490 nm, indicating the formation of excimers (Supplementary Figure 2), which is consistent with a previous study (Breukink et al., 2003). The addition of brevibacillin or brevibacillin 2V only decreased the monomer fluorescence of pyrene-labeled Lipid II, but did not bring Lipid II molecules together as no excimer fluorescence could be detected (Figures 6A,B). These results again demonstrate that brevibacillins can bind with Lipid II, but do not recruit Lipid II molecules in an oligomeric cluster.

### Brevibacillins Disrupt the Membrane Without the Need for Lipid II-Binding *in vitro*

To investigate the role of Lipid II-binding ability of brevibacillins in their membrane disruption capacity, CF leakage assays were performed using LUVs composed of DOPC with or without Lipid II and containing CF at self-quenching concentrations. LUVs-based CF leakage assay is widely used in the studies of the antibiotic mode of action (Hasper et al., 2004; Smith et al., 2008; Slootweg et al., 2015; Bakhtiary et al., 2017). Disruption of the vesicle membrane by certain compounds releases CF from the LUVs into the extra-vesicular milieu resulting in increased fluorescence signal (Figure 7A). In the Lipid II containing LUVs, both brevibacillin and brevibacillin 2V caused rapid membrane permeabilization (Figure 7B). In addition, brevibacillin caused a higher percentage of CF leakage than brevibacillin 2V. In LUVs lacking Lipid II, brevibacillin and brevibacillin 2V still caused rapid disruption of membrane. Moreover, brevibacillins caused a lower percentage of CF leakage in the presence of Lipid II (Figures 7B,C), indicating that some of the brevibacillins were bound to Lipid II. These results suggest that membrane disruption and Lipid II binding of brevibacillins are two independent antimicrobial modes of action that both can contribute to their antimicrobial activity. Previous studies showed that some Lipid II-targeting antimicrobials, i.e., lacticin 3147 and HNP-1, cause membrane disruption in both Lipid II containing LUVs and Lipid II lacking LUVs (Varney et al., 2013; Bakhtiary et al., 2017). Thus, they appear to display a similar mode of action as brevibacillins. In addition, non-specific pore-formation was observed for many antimicrobial peptides at a μm concentration level (Oren and Shai, 1998; Huang et al., 2004; Brogden, 2005). Here, we show that brevibacillin and brevibacillin 2V cause the leakage of CF at a much lower concentration (100 nm) than their MIC value (1.3 μm).

To investigate the *in vivo* membrane disruption capacity of brevibacillin and brevibacillin 2V, a membrane permeability assay was performed with a series of concentrations of antibiotics. Nisin was used as a control. The results showed that nisin has a significantly (*p* < 0.05) higher membrane permeabilization capacity than the brevibacillins at concentrations of 100 nm and 400 nm (Figures 7D,E). In addition, brevibacillin showed a significantly (*p* < 0.05) higher membrane permeabilization capacity than brevibacillin 2V at concentrations of 100 nm and 400 nm (Figures 7D,E), which is consistent with the results of the *in vitro* membrane permeability assay (Figures 7B,C). However, to permeabilize virtually all cellular membranes, a similar concentration of brevibacillin and brevibacillin 2V is needed (Figures 3C, 7D,E). These results provide more insight into the mode of action of brevibacillins.

### Brevibacillins Likely Bind to the GlcNAc-MurNAc Moiety and/or Part of the Pentapeptide of Lipid II

**Figure 8:**
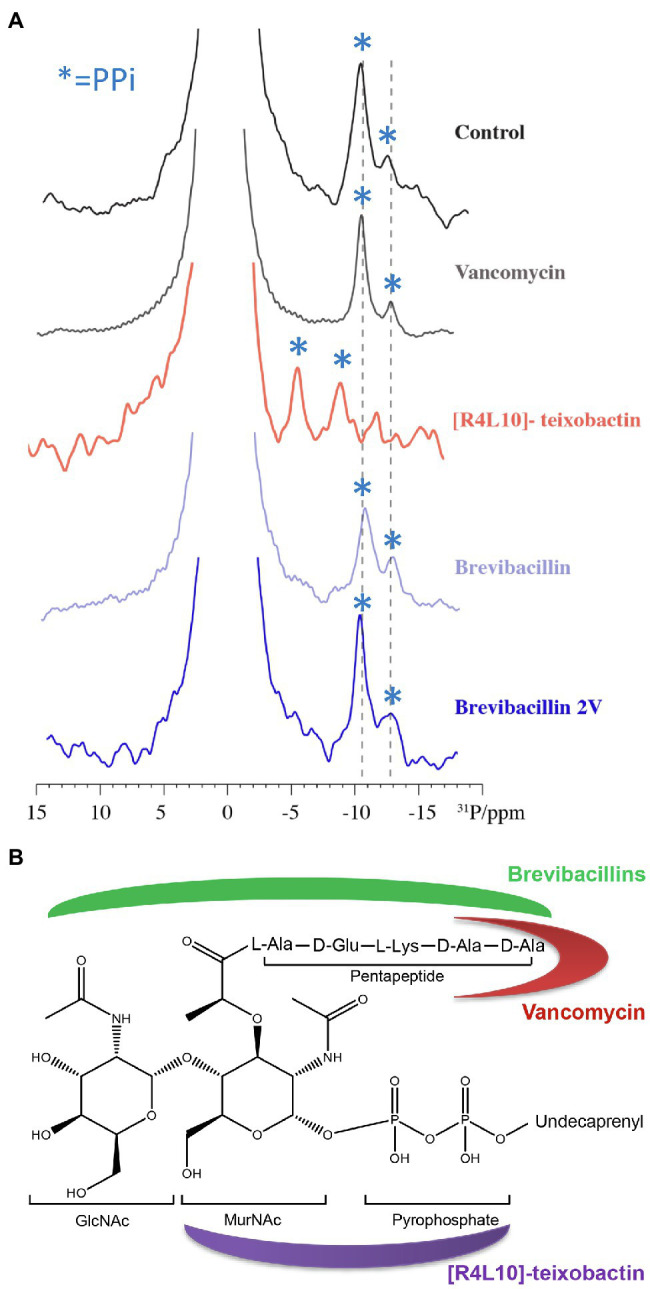
Brevibacillins likely bind to the GlcNAc-MurNAc moiety and/or the pentapeptide of Lipid II. **(A)**, 1D ^31^P spectra of Lipid II in liposomes in the presence of vancomycin, [R4L10]-teixobactin, brevibacillin, and brevibacillin 2V. Blue asterisks mark PPi signals; bulk lipids come around 0 ppm. **(B)**, Structure of Lipid II (Münch and Sahl, 2015; Shukla et al., 2020). Red moon indicates the Lipid II-binding site of vancomycin; purple moon indicates the Lipid II-binding site of [R4L10]-teixobactin; and green moon indicates the likely Lipid II-binding site of brevibacillins.

To investigate whether brevibacillins bind to the pyrophosphate moiety of Lipid II, ^31^P ssNMR assays were performed. [R4L10]-teixobactin was used as a positive control that binds to the pyrophosphate moiety of Lipid II (Shukla et al., 2020). In addition, vancomycin was used as a control that binds to part of the pentapeptide of Lipid II (Münch and Sahl, 2015). ^31^P ssNMR data show clear shifts of the Lipid II pyrophosphate moiety signals upon binding of [R4L10]-teixobactin (Figure 8A), indicating a direct interaction of pyrophosphate moiety and [R4L10]-teixobactin, which is consistent with a previous study (Shukla et al., 2020). However, there were no changes observed for the Lipid II pyrophosphate signals treated with either vancomycin or any of the brevibacillins (Figure 8A). The ^31^P ssNMR data suggest that brevibacillins do not directly interact with the pyrophosphate moiety of Lipid II. In addition, due to the fact that the undecaprenyl tail of Lipid II is embedded in the membrane, the ^31^P ssNMR data results suggest that brevibacillins likely bind to the GlcNAc-MurNAc moiety and/or pentapeptide of Lipid II. Due to the production of modified Lipid II, which has a modified pentapeptide (L-Ala-D-Glu-L-Lys-D-Ala-D-Lac) instead of the wide-type pentapeptide (L-Ala-D-Glu-L-Lys-D-Ala-D-Ala), many bacterial pathogens became resistant against vancomycin, such as *Enterococcus faecium* (VRE), *Enterococcus faecalis* (VRE), and *S. aureus* (VRSA; Arthur et al., 1996; Lebreton et al., 2011; Münch and Sahl, 2015). Previous studies reported that brevibacillins have potent antimicrobial activity against both vancomycin-resistant and other susceptible bacterial pathogens (Yang et al., 2016; Wu et al., 2019; Zhao et al., 2021), suggesting that brevibacillins have a different mode of action with vancomycin. These results point to a mechanism where brevibacillins likely bind to the GlcNAc-MurNAc moiety and/or pentapeptide of Lipid II (Figure 8B).

## Conclusion

Here, we show that brevibacillin 2V acts as a bactericidal antimicrobial agent against *S. aureus* (MRSA). Further studies demonstrate that brevibacillin 2V exerts its bactericidal activity by binding to the bacterial cell wall synthesis precursor Lipid II and permeabilizing the bacterial membrane. In addition, our results suggest that the membrane disruption capacity and Lipid II-binding motif of brevibacillins are two independent antimicrobial modes of action, which is good for avoiding drug resistance development (Martin II et al., 2020). Combined ssNMR, CD, and ITC assays indicate that brevibacillin 2V binds to the GlcNAc-MurNAc moiety and/or the pentapeptide of Lipid II. Compared to other membrane and Lipid II targeting peptides, such as nisin (Hasper et al., 2006), lacticin 3147 (Wiedemann et al., 2006), and haloduracin (Oman et al., 2011), brevibacillin 2V has a much lower molecular weight, which might make it relatively easier to reach the infection site *in vivo*. In addition, brevibacillin 2V has shown good stability in human plasma due to the presence of non-canonical amino acids and D-amino acids (Zhao et al., 2021). This study provides a valuable insight into the antimicrobial mode of action of brevibacillin 2V. As brevibacillin 2V is a novel and promising antibiotic candidate with low hemolytic activity and cytotoxicity, the here-elucidated mode of action will help further studies that could develop it as an alternative antimicrobial agent.

## Data Availability Statement

The original contributions presented in the study are included in the article/Supplementary Material, further inquiries can be directed to the corresponding author.

## Author Contributions

OK and XZ conceived the project. XZ designed and carried out the experiments, analyzed the data, and wrote the manuscript. OK and EB supervised the work and corrected the manuscript. XW, RS, RK, and MW did experimental work on NMR and Lipid II binding. All authors contributed to and commented on the manuscript text and approved its final version.

### Conflict of Interest

The authors declare that the research was conducted in the absence of any commercial or financial relationships that could be construed as a potential conflict of interest.

## Supplementary Material

The Supplementary Material for this article can be found online at https://www.frontiersin.org/articles/10.3389/fmicb.2021. 694847/full#supplementary-material
