# Supplementary material for: Brevibacillin 2V Exerts Its Bactericidal Activity via Binding to Lipid II and Permeabilizing Cellular Membranes
Source: Front Microbiol. 2021 Jul 16;12:694847. doi: 10.3389/fmicb.2021.694847 (PMC8322648; doi:10.3389/fmicb.2021.694847)
Supplement: Supplementary file 1 [file Data_Sheet_1.DOC]

Supplementary material

Brevibacillin 2V exerts its bactericidal activity via binding to Lipid II and permeabilizing cellular membranes

Xinghong Zhao ^1^, Xiaoqi Wang ^2^, Rhythm Shukla ^2, 3^, Raj Kumar ^3^, Markus Weingarth ^3^, Eefjan Breukink ^2^, Oscar P. Kuipers ^1^, *

^1^ Department of Molecular Genetics, Groningen Biomolecular Sciences and Biotechnology Institute, University of Groningen, Nijenborgh7, 9747 AG Groningen, The Netherlands.

^2^ Membrane Biochemistry and Biophysics, Bijvoet Centre for Biomolecular Research, Department of Chemistry, Faculty of Science, Utrecht University, Padualaan 8, Utrecht, The Netherlands.

^3^ NMR Spectroscopy, Bijvoet Centre for Biomolecular Research, Department of Chemistry, Faculty of Science, Utrecht University, Padualaan 8, Utrecht, The Netherlands.

* Correspondence: o.p.kuipers@rug.nl (Oscar P. Kuipers)


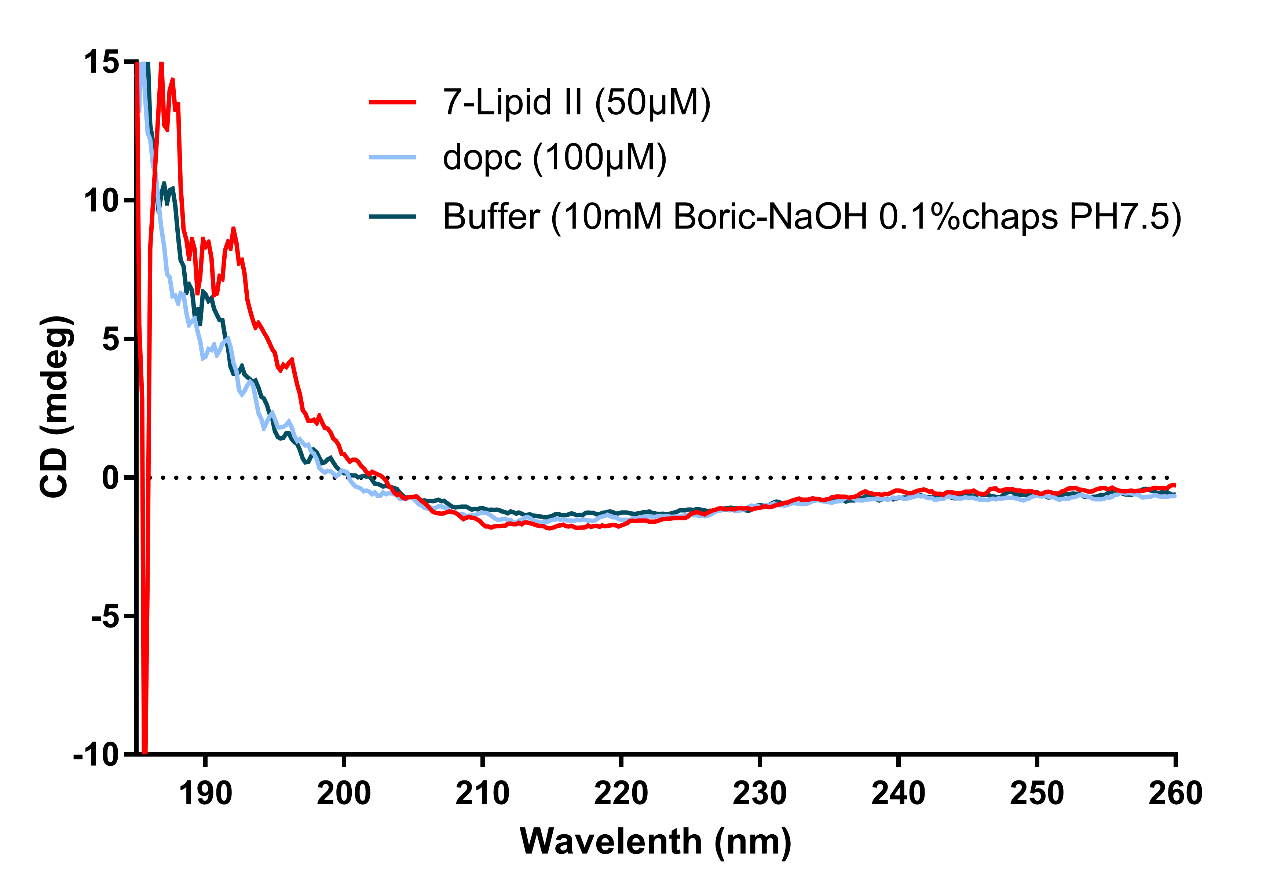


**Supplementary Figure 1.** CD spectrum controls for 7-Lipid II binding.


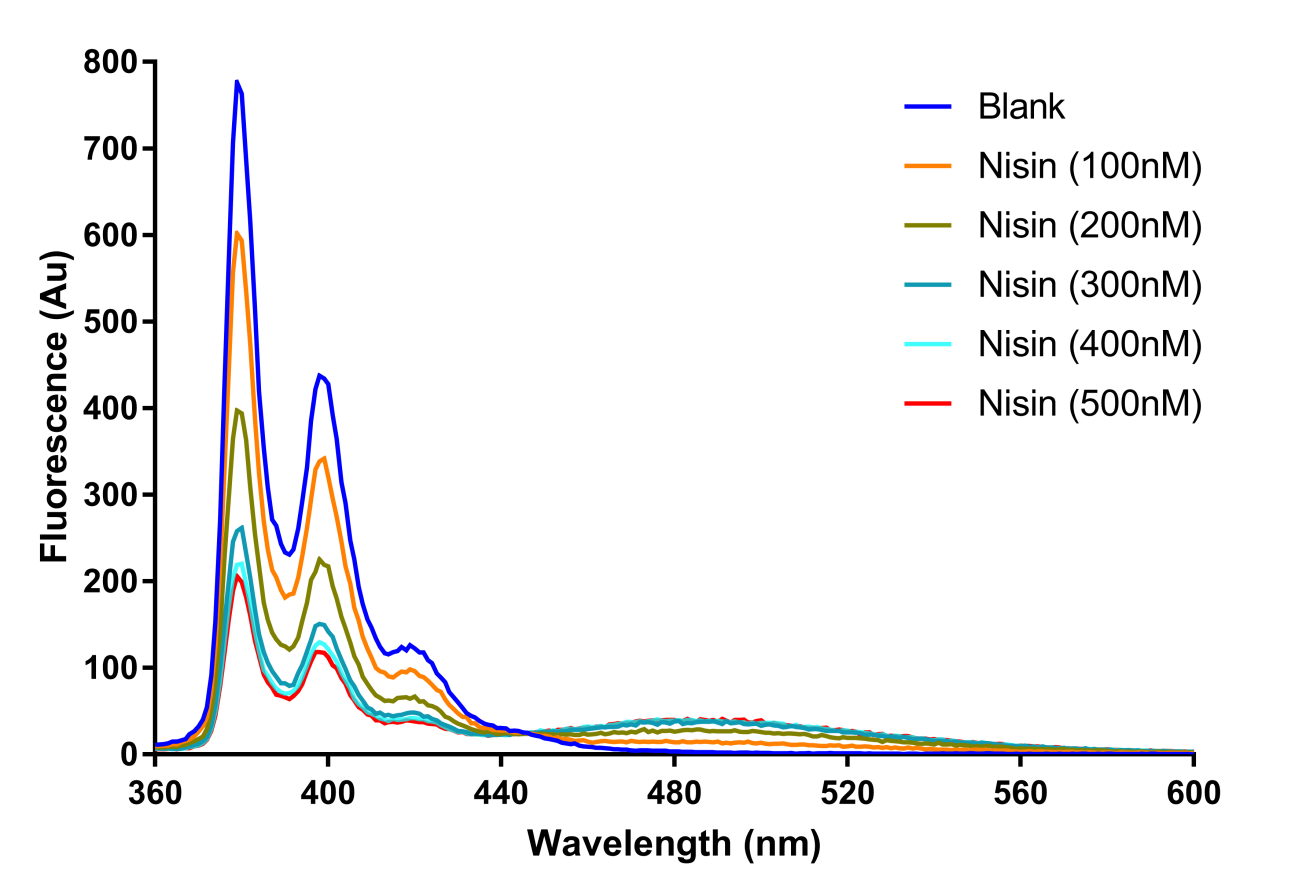


**Supplementary Figure 2.** Effect of increasing nisin concentrations on the fluorescence characteristics of pyrene-labeled Lipid II at 0.5 mol % in DOPC bilayers.
